# Perceived contrast on displays with different luminance ranges

**DOI:** 10.1002/mp.15519

**Published:** 2022-02-15

**Authors:** Patrik Sund

**Affiliations:** ^1^ Department of Medical Physics and Biomedical Engineering Sahlgrenska University Hospital Göteborg Sweden; ^2^ Institute of Clinical Sciences University of Gothenburg Göteborg Sweden

**Keywords:** image quality, medical display, visual perception

## Abstract

**Purpose:**

Medical displays are fundamental in today's healthcare since they provide the link between digitally stored data and the human clinician, and it is thus important that the transfer of information is as effective and reliable as possible. Contrast perception in viewed images is complex due to the nature of the human visual system, and the luminance distribution in the viewed scene plays a major role. Standards and guidelines concerning medical displays are important as they set a baseline image quality. However, as the number of imaging applications as well as display technology has evolved rapidly during the past decades, there may be possible uses not foreseen in the current guidelines. Bright screens may perform as good in bright rooms as less bright displays do in dark rooms, but current guidelines are likely to favor dark rooms due to historical reasons. The purpose of this study was to determine the limits of contrast perception in three very different lighting conditions and relate the outcome to guideline recommendations.

**Methods:**

Three different display luminance settings were studied, 1–250, 6–500, and 12–750 cd/m^2^ with luminance ratios of 250, 85, and 62, respectively. Although the luminance ratios, black levels, and white levels were different, they all covered the same number of just noticeable differences (JNDs). By using a two‐alternative forced‐choice method, contrast thresholds were determined at dark, mid‐gray, and bright pixel values for all luminance settings using bar patterns with two different spatial frequencies. In total, 18 contrast thresholds were determined by each of the 10 observers.

**Results:**

The contrast thresholds for the low‐frequency patterns were close to 0.5 JNDs and there were no systematic differences between the three luminance conditions at any of the pixel values. The high‐frequency patterns required almost 10 times higher contrast where the highest contrast threshold (worst visibility) was obtained for the luminance setting 1–250 cd/m^2^ at the dark pixel value.

**Conclusions:**

The differences between the three luminance conditions were mostly minor, which indicate that display settings with low luminance ratios and high minimum luminance levels can be used without compromising displayed image contrast. The number of JNDs enclosed by the luminance range of a display is a reliable metric for global perceived contrast. Luminance ratios are limited regarding the ability to detect low contrast objects when there are large differences in luminance, although they can still be used within a relatively small range of luminance levels. Low luminance levels may cause a loss of visibility, especially for fine details, and should be avoided.

## 1 INTRODUCTION

Quality assurance of medical displays is important for consistent and optimal rendition of clinical images. The display is the link that translates digital pixel values into visible light perceivable by the human visual system (HVS). Standardized display properties ensure adequate quality of rendered medical images. There are numerous standards and guidelines that specify display requirements like, for example, display size, resolution, homogeneity, noise, and temporal performance.^1^, ^2^, ^3^, ^4^, ^5^, ^6^, ^7^, ^8^, ^9^, ^10^ This paper will focus on the validity and limitations of the requirements for display luminance range, that is, the minimum and maximum luminance, and the distance between them.

The first displays to be used in medical imaging had a low maximum luminance and a high reflectance.^11^ The most logical thing to do was to use them in dark rooms where reflections were reduced, luminance ratio increased, and contrast improved. It was also a simple and natural solution to use the minimum and maximum luminance together with the corresponding luminance ratio as display requirements. The technical aspects of displays have improved rapidly since then. Today, the maximum luminance can be very high and reflectance very low, which allows high‐contrast renditions in much brighter environments, such as operating rooms and dentist departments.

For medical applications, the most commonly used requirement for display input to luminance output characteristics is the grayscale standard display function (GSDF) in Dicom part 14.^12^ This function utilizes the concept of just noticeable differences (JNDs) to distribute the perceived contrast evenly throughout the entire luminance range. In short (and somewhat simplified), the number of JNDs enclosed by the minimum and maximum luminance of a display corresponds to the number of theoretically visible gray levels.

The luminance ratio functions reasonably well in dark environments, but a display with a high minimum luminance requires the maximum luminance to exceed unrealistic levels if the specified luminance ratio is also high. In this case, the number of JNDs is probably a better requirement than the luminance range for specifying display contrast since it accounts for the nonlinear nature of the HVS. As an example (see Tables 1 and 2), consider the following recommendations for diagnostic displays according to AAPM TG270: Minimum luminance 1 cd/m^2^; maximum luminance 350 cd/m^2^; luminance ratio 350.^2^ The corresponding JND range is 582 and will in this example be used as an alternative to the luminance ratio requirement. In a brighter room where the display minimum luminance is 4 cd/m^2^, 582 JNDs can be achieved with a maximum luminance of 578 cd/m^2^ while a luminance ratio of 350 would require the maximum luminance to be 1400 cd/m^2^, which is well above the specifications of most diagnostic displays. Even in a very bright environment, a high‐end medical display would be possible to use with a luminance range of 6–700 cd/m^2^. In this case, the JND range is still 582 but the luminance ratio is only 117.

**TABLE 1:** Possible luminance ranges for a display with a luminance ratio of 350

Minimum luminance (cd/m^2^)	Luminance ratio	Maximum luminance (cd/m^2^)	JND range
1	350	350	582
2	350	700	652
4	350	1400	716
6	350	2100	749

**TABLE 2:** Possible luminance ranges for a display with a JND range of 582

Minimum luminance (cd/m^2^)	JND range	Maximum luminance (cd/m^2^)	Luminance ratio
1	582	350	350
2	582	437	218
4	582	578	144
6	582	700	117

A fixed luminance ratio thus requires the minimum luminance to be relatively low to keep the maximum luminance within a realistic range, while a fixed JND range would allow diagnostic displays to be used in much brighter environments. Image quality can be critical also in bright environments where dimming of the lights is not possible. Unfortunately, displays in bright rooms tend to be of low cost with little or no quality control. Quality displays with QA capabilities are more expensive than standard displays and the investment is difficult to justify given that, at best, only lower requirements (review displays) can be fulfilled. However, if the assumption of equal perceived contrast for displays with equal JND ranges is valid, the actual image quality can be just as good in bright rooms as in dark rooms. Today, higher requirements (diagnostic displays) are impossible to meet in bright rooms due to the luminance ratio requirement that forces the maximum luminance to unrealistic levels. For the end users in bright locations, there are no guidelines on how to best use their displays. For a more comprehensive theoretical study concerning luminance ranges and possible strategies for maintaining stable image contrast in bright rooms, please see another recently published paper by Sund.^13^

Replacing luminance ratios with JND ranges would not only allow standardized display properties in environments that cannot be dimmed, but it would also be possible to use much brighter reading rooms in general. Both AAPM Report 270 and ACR‐AAPM‐SIIM recommend that the minimum luminance is not too low to avoid the mesopic region of the HVS.^2^, ^8^ The HVS performs better with more light.^14^, ^15^ Fatigue may also be reduced with more light.^16^ There are a few detection studies related to the visibility of images under different lighting conditions. Pollard showed that a moderate increase in illuminance (<100 lx) will likely not degrade, and may even improve, an observer's ability to detect low‐contrast objects provided that the luminance ratio is maintained.^17^, ^18^, ^19^ Other studies demonstrated that an increase in ambient light will degrade the visibility of image details.^20^, ^21^, ^22^ However, the increase in ambient light was never accompanied by a corresponding increase in the display's maximum luminance, thereby reducing both the luminance ratio and the JND range.

The purpose of this study was to determine the limits of contrast perception in three very different lighting conditions and relate the outcome to guideline recommendations. A human observer study will determine the related contrast thresholds using a two‐alternative forced‐choice (2AFC) method.

## 2 MATERIALS AND METHODS

### 2.1 Terminology

*L*_min_: Minimum luminance output from a display (cd/m^2^).
*L*_max_: Maximum luminance output from a display (cd/m^2^).
*E*: Room illuminance measured at the display surface when the display is off (lx).
*R*_d_: Display diffuse reflection coefficient (cd/m^2^/lx).
*L*_amb_: Reflected luminance from the display (cd/m^2^). [*L*_amb_ = *E* × *R*_d_]
*L*′_min_: Minimum luminance output from a display including reflected light (cd/m^2^). [*L*′_min_ = *L*_min_ + *L*_amb_]
Also referred to as “Black level.”
*L*′_max_: Maximum luminance output from a display including reflected light (cd/m^2^). [*L*′_max_ = *L*_max_ + *L*_amb_]
Also referred to as “White level.”
*r*′: Display luminance ratio. [*r*′ = *L*′_max_/*L*′_min_]

### 2.2 Equipment

A high‐end medical display (Eizo Radiforce RX350, Eizo, Hakusan, Japan) utilizing a display board capable of 10‐bit rendering (FirePro W5100, AMD, Sunnyvale, CA, USA) was placed in a small room without windows where the walls and ceiling were painted in a matte black color and the gaps in the door framing were covered with black plastic sheets. The ambient light sources consisted of three Philips Hue color ambience E27 lights (Philips Lighting, Eindhoven, The Netherlands) positioned close to the ceiling, directly above the observer. The display luminance output was measured using an LS‐100 telescopic luminance meter (Konica Minolta, Tokyo, Japan). All display calibrations and observer performance studies were made using the same software developed in‐house by the author. During all luminance measurements, conditions such as display brightness, display mode, internal look‐up‐tables (LUTs), graphic board LUTs, and room lights properties were always recorded. The ambient light was also measured using the illuminance meter in the bezel of the display. Illuminance at screen center was measured using a Hagner Universal photometer model S2 (Hagner, Solna, Sweden).

### 2.3 Display calibration

Three different luminance and illuminance settings were used as shown in Table 3. They all covered 533 JNDs but had vastly different luminance ratios. The black and white levels were set at calibration by adjustment of the display brightness and internal LUT. The purpose of using higher illuminances for higher luminance settings was to reduce the possible eye strain caused by viewing a bright display in a dark room. The calibrations were made from *L*′_min_ to *L*′_max_ according to Dicom part 14 using the specified test pattern and 256 measurement points. Calibrations were stored in the display internal LUT and the LUT on the display board was linear. For each luminance setting, the maximum luminance of the uncalibrated display was adjusted to be somewhat higher than *L*_max_ when calibrated. By keeping the maximum luminance of the uncalibrated display only slightly higher than when calibrated, the loss of luminance resolution caused by a partially used LUT was minimized.

**TABLE 3:** Luminance settings used in the observer studies

Luminance setting	*L*′_min_ (cd/m^2^)	*L*′_max_ (cd/m^2^)	*E* (lx)	*L*_amb_ (cd/m^2^)	*r*′	#JND	Room lights
Low	1.0	250	0.4	0.002	250	533	off
Medium	5.9	500	27	0.14	85	533	50 %
High	12.1	750	110	0.55	62	533	100 %

Notes: L′_min_ is the minimum luminance including reflected light. L′_max_ is the maximum luminance including reflected light. E is the illuminance at the center of the display surface when the display is off. L_amb_ is the luminance caused by reflected light. r*′* is the ratio between L*′*_max_ and L*′*_min_. #JND is the number of JNDs enclosed by L*′*_min_ and L*′*_max_. R_d_ is assumed to be 0.005 cd/m^2^/lx.

### 2.4 Test image

**FIGURE 1:**
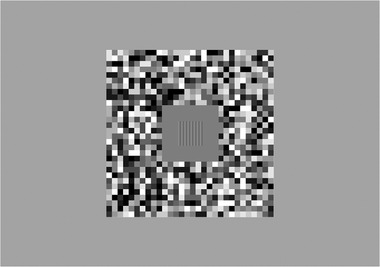
Test image for luminance measurements and test pattern observations. The center bar pattern was only present during the observer studies and not during luminance measurements. In this image, the bar pattern contrast has been greatly exaggerated for demonstration purposes. Normally, it was barely visible

The test image used (see Figure 1) was the same for both the display luminance measurements and the observer studies. The intention of the test image was to simulate the luminance variations in a typical X‐ray image. The entire screen was set to pixel value 127 using an 8‐bit gray‐scale rendering, corresponding to mid‐gray. In the center 900 × 900 pixels (19 × 19 cm), the actual test image was displayed using a 10‐bit OpenGL rendering technique. The test image was divided into 30 × 30 squares where the pixel values were generated only once, in the beginning of the study, by a random selection from 11 pixel values uniformly distributed between 0 and 1023. The center 10 × 10 squares were then replaced by a homogenous area that could take any pixel value. During the observer studies, but not during the luminance measurements, a low‐contrast bar pattern (64 × 64 pixels) was displayed in the center of the image, where the bar pattern average luminance was the same as the luminance of the surrounding center homogenous area.

### 2.5 Luminance measurements and test pattern generation

Measuring luminance output valid for the actual viewing conditions was not trivial, even with a high‐end telescopic luminance meter. The major problem concerned the lowest luminance output from the display. Even though the room lights were off, light from the bright parts of the image was reflected in the luminance meter and sent back to the screen surface, thereby increasing reflected light. The distance of the luminance meter to the display greatly influenced the measured values. To achieve a measuring geometry valid for when an observer is viewing images, the luminance meter was placed approximately at the position where the observer's head was supposed to be.

The Minolta LS‐100 has a circular measurement area covering a 1° viewing angle while the viewfinder covers 9°. The influence of light from the viewfinder outside the measurement area is normally small but can be substantial when measuring a small dark area surrounded by bright regions.^23^ The normal closest focusing distance of the luminance meter is 1 m, but due to the head‐simulating position closer to the screen, a close‐up lens (lens 135) had to be used. Due to light attenuation in the lens, all measured values were multiplied with 1.05, as specified by the Minolta user manual. Using this lens had another positive effect—the entire viewfinder field of vision was within the boundaries given by the center homogenous area of the test image, thereby reducing the effect of bright light from outside the measurement area.

Since extremely low‐contrast test patterns (down to 0.1 JND) were to be displayed at different luminance levels, it was crucial to measure the display luminance response at the highest possible luminance resolution. An 8‐bit display covering 533 JNDs has a luminance resolution of 2.1 JNDs per pixel value change while a 10‐bit display has 0.5. Since even a 10‐bit display would be insufficient, any of the three colored subpixels could deviate by one pixel value from the other two, thereby increasing the number of (near) gray levels to 7162 (0.074 JND per subpixel change).^24^, ^25^ The luminance response for each of the three luminance settings was measured three times and the average value for each gray‐level was used for test pattern generation.

Since the distances between subsequent luminance values were irregular, the software searched for the best possible test pattern with a given luminance and contrast, within given tolerances (±5% for luminance and ±0.01 JND for contrast). The requirement for a good bar pattern was that the higher and lower luminance levels were equally spaced from a center luminance level. This center luminance level was then used for the homogenous area in which the bar pattern was positioned. By using this method, any difference in bar pattern average luminance from the luminance in the surrounding homogenous area was too small to be detectable.

### 2.6 Observer studies—Determination of contrast thresholds

For each of the three luminance settings, contrast thresholds were determined for bar patterns at three pixel values: 50 (dark), 500 (mid‐gray), and 950 (bright). Two different bar pattern spatial frequencies were also used, one with 8 pixels per period (4 high + 4 low) and one with 2 pixels per period (1 high + 1 low). In total, 18 contrast thresholds were determined for each observer using a 2AFC technique together with an adaptive method in what is referred to as a run.^26^ Each run took approximately 10 min to complete, and the observers were free to decide the number of consecutive runs in each session before breaking. Before each new run, the observers had to wait at least 30 seconds for their vision to adapt and for the display and room lighting to stabilize. The order of the runs was randomized for each observer, but all lower frequency patterns were always viewed before any of the higher frequency patterns. All technical aspects associated with each run, such as display luminance, display mode, LUTs, room light level settings, and room illuminance, were automatically set and verified by the software before each run could start. The viewing distance was 39 cm and for the 8 pixels per period patterns, this corresponded to the standard target defined by Dicom part 14 (A 2‐deg × 2‐deg square filled with a horizontal or vertical grating with sinusoidal modulation of 4 cycles per degree), apart from the fact that bar patterns were used instead of sinusoidal patterns.

**FIGURE 2:**
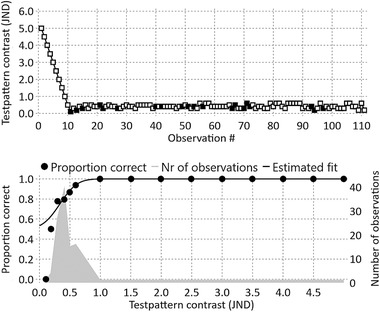
Results from one run, that is, the determination of one contrast threshold for one observer. In the upper chart, test pattern contrast is shown for every observation in the run. White squares indicate correct answers while black squares indicate incorrect answers. The lower chart shows the proportion of correct observations for each test pattern contrast together with the best possible fit for the modified cumulative gaussian function. The number of observations for each contrast level is represented by the filled gray area. The contrast threshold was set to the test pattern contrast resulting in 75% correct answers according to the fitted function

There are many possible methods to determine contrast thresholds.^27^, ^28^, ^29^, ^30^, ^31^, ^32^, ^33^ The one used in this study utilized 2AFC together with an adaptive method that determined the contrast of the upcoming pattern in the run based on previous answers.^26^ The goal for the adaptive method was to choose patterns with a contrast close to, or slightly above, the estimated contrast threshold. For each of the 18 setups, a set of bar patterns was created within a plausible contrast range, considering that different observers have different contrast sensitivity. Each bar pattern could be displayed in one of two directions, vertical or horizontal, and the observer had to decide the most likely direction. The first pattern was always the one with the highest contrast and the following patterns had decreasing contrast until the observer made an erroneous decision. From that point on, the contrast of the next pattern was always determined by using all the observer's previous responses combined with a modified version of the cumulative Gaussian distribution. The modified version spanned from 0.5 to 1.0 since the guess rate in a 2AFC study is 50%. The best least squares adaptation of the modified cumulative Gaussian to all responses was determined using a Cobyla optimization algorithm. After 100 observations from the first error, the psychometric curve was approximated by the last fit of the modified cumulative Gaussian function. The contrast threshold was determined by the mean value in the Gaussian distribution, corresponding to a response rate of 75% correct observations, which is midway between guessing and 100% correct. See Figure 2 for an example run.

Each contrast threshold mean value was calculated by averaging the individual contrast thresholds for the ten observers. Confidence intervals and paired difference tests were calculated using bootstrapping.^34^, ^35^, ^36^ For each bootstrap sample, ten observers were randomly selected (with replacement), making the results representative of a general population of observers. For each of the observers’ runs, the observations at all test pattern contrasts were replaced by bootstrapping from the original observations at that contrast level, thus resulting in new psychometric curves and new contrast thresholds. 2000 bootstrap samples were performed, resulting in the same number of mean value estimations for all contrast thresholds. The confidence interval for each mean was calculated as the middle 95% of all estimations. Difference distributions were calculated by creating pairwise differences between all contrast threshold estimations. The *p*‐value related to two contrast thresholds, indicating the probability that the two thresholds were equal, was determined by the position of zero within the difference distribution. If zero splits the distribution exactly in the middle, there is (on average) no difference resulting in a *p*‐value of 1. If zero splits the distribution with 2.5% of all values on one side and 97.5% on the other side, the *p*‐value is 0.05.

## 3 RESULTS

18 contrast thresholds were determined by each of the 10 observers in a total of 180 2AFC runs. The result from one of the runs is shown in Figure 2. Most of the observations were made close to the detection threshold.

**FIGURE 3:**
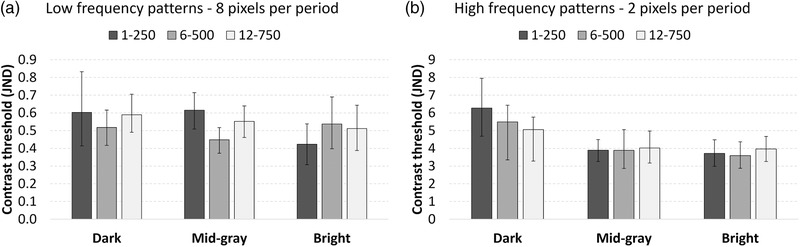
Average contrast thresholds for the ten observers. Left chart (A): Low‐frequency patterns. Right chart (B): High‐frequency patterns. Three display luminance conditions were studied, 1–250, 6–500, and 12–750 cd/m^2^. For each condition, contrast thresholds were determined at three pixel values: dark (50), mid‐gray (500), and bright (950). Error bars indicate the 95% confidence intervals

The average contrast thresholds for the ten observers are shown in Figure 3 for two bar pattern frequencies, three luminance conditions and three pixel values. Pair‐wise *p*‐values indicating the probability that two contrast thresholds are equal are shown in Table 4.

**TABLE 4:** Pair‐wise p‐values indicating the probability that the contrast thresholds are equal. The top right half contains values for the high‐frequency patterns while the lower left half contains values for the low‐frequency patterns. Values below 0.05 are bold

	1–250 Dark	1–250 Mid‐gray	1–250 Bright	6–500 Dark	6–500 Mid‐gray	6–500 Bright	12–750 Dark	12–750 Mid‐gray	12–750 Bright	
**1–250 Dark**		**<0.01**	**<0.01**	0.068	**<0.01**	**<0.01**	**0.024**	**<0.01**	**<0.01**	**1–250 Dark**
**1–250 Mid‐gray**	0.847		0.587	0.201	0.967	0.408	0.519	0.711	0.865	**1–250 Mid‐gray**
**1–250 Bright**	0.056	**<0.01**		0.08	0.658	0.774	0.364	0.162	0.466	**1–250 Bright**
**6–500 Dark**	0.339	0.062	0.116		0.122	0.052	0.575	0.225	0.27	**6–500 Dark**
**6–500 Mid‐gray**	0.132	**<0.01**	0.552	0.163		0.406	0.512	0.719	0.908	**6–500 Mid‐gray**
**6–500 Bright**	0.477	0.161	0.057	0.933	0.179		0.238	0.158	0.422	**6–500 Bright**
**12–750 Dark**	0.959	0.566	**<0.01**	0.078	**<0.01**	0.395		0.721	0.658	**12–750 Dark**
**12–750 Mid‐gray**	0.666	0.259	**0.01**	0.492	**0.016**	0.694	0.582		0.824	**12–750 Mid‐gray**
**12–750 Bright**	0.446	0.053	0.052	0.973	0.186	0.878	0.268	0.54		**12–750 Bright**
	1–250 Dark	1–250 Mid‐gray	1–250 Bright	6–500 Dark	6–500 Mid‐gray	6–500 Bright	12–750 Dark	12–750 Mid‐gray	12–750 Bright	

The contrast thresholds for the low‐frequency patterns were close to 0.5 JNDs for all luminance settings and pixel values, while the contrast thresholds for the high‐frequency patterns were almost 10 times higher.

The GSDF is based on the visibility of test patterns with a frequency corresponding to the lower frequency used in this study. For this frequency, the contrast thresholds at all three pixel values were, with a few exceptions, almost the same and thereby close to perceptually linear. Although there were some statistically significant differences, they were not systematic. The issue of possible systematic errors (which are not reflected in the *p*‐values) when determining very low‐contrast thresholds is addressed in the discussion. For the high‐frequency patterns, no significant differences could be found between the three luminance settings at the mid‐gray and bright pixel values. However, at the dark pixel values, the contrast threshold for 1–250 cd/m^2^ was significantly higher than for 12–750 cd/m^2^. The contrast threshold for 1–250 was also significantly higher at dark pixel values than at mid‐gray and bright pixel values. For the other high‐frequency pattern luminance settings, there were no significant differences between any of the pixel values.

## 4 DISCUSSION

The JND is based on human perception studies under different lighting conditions, and it is not surprising that the JND range of a display is a valid metric for perceived contrast. In this study, contrast properties for displays with three different luminance ranges, but with the same JND range, were examined. The differences were found to be minor with similar contrast properties at the three pixel values: Dark, mid‐gray, and bright. Although there were a few statistically significant differences between the luminance settings for the low‐frequency patterns, there was no trend indicating which setting performed best. The differences are likely indications of the difficulties involved when determining extremely low‐contrast thresholds. For example, one of the used test patterns had a contrast of 0.51 JND where the maximum luminance was 31.353 cd/m^2^ and the minimum luminance was 31.213 cd/m^2^. The corresponding pixel values [red, green, blue] were [499,498,498] and [498,497,498], respectively. With such small differences in luminance, measurement uncertainties and display stability may introduce small systematic errors.

The GSDF accounts for different contrast thresholds at different luminance levels, but only for the specific frequency corresponding to the low‐frequency patterns in this study. The higher contrast thresholds obtained in dark image areas for high‐frequency patterns is an indication of the theoretical fact that small details are difficult to see when the luminance is low.^37^ Image quality would probably improve by avoiding low display luminance levels. Another possible reason could be because the luminance ratio is highest for 1–250 cd/m^2^ and lowest for 12–750 cd/m^2^. A high luminance ratio causes a decrease in contrast sensitivity as the difference between image object luminance and adaptation luminance increases.^37^, ^38^, ^39^, ^40^ Although the GSDF was a big step toward perceptual linearization, the JND is only valid when viewing a single luminance at a time.^12^ One solution is to use a modified version of the GSDF that takes the luminance range into account.^40^ If an unmodified version of the GSDF is used, a smaller luminance range may even be beneficial for the perceived contrast since the HVS operates closer to peak contrast sensitivity.^13^

A common misconception about image display is that a large luminance range with a dark black and a bright white is always better than a low luminance range. The HVS has a remarkable capability of scaling a multitude of luminance ranges into the same perceived gray‐scale range.^41^ The risk of experiencing dull and gray images on a display with a low luminance range is therefore low, provided that the JND range is sufficiently high. Medical display calibration is about contrast visibility, and the spacing between gray‐levels is far more important than their actual intensity.

The measured contrast thresholds in this study, expressed in JNDs, reflect properties of the HVS, and do not depend on the number of JNDs per gray level. However, when viewing images, a large JND range is important for low‐contrast visibility, and a large number of gray levels (image and display bit depth) is important to reduce luminance quantization effects. The minimum requirements for these parameters are probably task dependent and outside the scope of this paper.

Consensus for the past 30 years has been to use a relatively low black level and a high luminance ratio, which is also the recommendations in most guidelines in the field of medical display. The historical reason behind these recommendations is that they provided an easily implementable solution for the two major problems of contemporary displays—high reflectance and low maximum luminance. Today, modern displays are very bright and use antireflective technology and are therefore much less problematic. Although the current recommendations still fulfill their purpose of excluding scenarios with poor image quality, they are also somewhat blunt, and exclude other scenarios with adequate or possibly even superior image quality. There is a possibility that some of the excluded scenarios will show increased image quality compared to the ones included, especially when the luminance increases to levels where the HVS perform better.

A display with a black level of 12 cd/m^2^ and a luminance ratio of 62 may seem unlikely to be used in a clinical environment, but when using a display with a reflection coefficient of 0.005 cd/m^2^/lx in an operating room with 2000 lx, this is close to reality. Another problem occurs with surgical light that has a central illuminance of 40 000–160 000 lx. To avoid adaptation problems, both the room illuminance and display luminance should be deliberately high. As the results from this study show, common diagnostic displays could very well be used under such conditions without compromising perceived contrast in any part of the image, if properly calibrated according to room illumination.

According to the visual model published by Barten,^37^ for a given luminance, higher spatial frequencies require higher contrast to be visible, which agrees with the results from this study. However, the expected increase in contrast between the two used frequencies is less than the obtained result, which was close to a factor of ten. One contributing factor could be pixel bleeding from the thin bright lines to the thin dark lines in the test pattern, thereby reducing the actual contrast. There is also a difference in luminance conditions between the model and this study. The model assumes ideal viewing conditions, whereas in this study, the pattern was positioned in the center of an area with large luminance variations. Intraocular light scattering is known to reduce the contrast sensitivity.^42^, ^43^

The rapid improvement in display technology with less reflective surfaces and higher light output has enabled the use of medical displays in bright environments with adequate image quality. Replacing the luminance ratio requirement with JND range would allow consistent image presentation for a wider range of operating conditions, including bright rooms. The possibility to use brighter displays in brighter rooms is not only of interest when dimming the lights is not feasible. Since the HVS is likely to perform better with more light, and humans in general tend to dislike working in dimly lit rooms, maybe it is time for radiologists to replace dark reading rooms with brighter locations. If the room illumination is constant and the display is calibrated accordingly, the perceived contrast will be as good, or possibly better, as in dark rooms.

## 5 CONCLUSIONS

The number of JNDs enclosed by the luminance range of a display is a reliable metric for global perceived contrast. Luminance ratios are limited regarding the ability to detect low‐contrast objects, since they do not take the properties of the HVS into account, although they can still be used within a relatively small range of low luminance levels. If the JND range requirements of a display are met, and the display is calibrated correctly according to ambient illumination, low luminance ratios and high black levels are possible to use without compromising image contrast.

## FUNDING INFORMATION

The author received no specific funding for this work.

## CONFLICT OF INTEREST

The author has no relevant conflicts of interest to disclose.

## Data Availability

Data available on request from the authors.

## References

[1] Samei E , Badano A , Chakraborty D , et al. Assessment of display performance for medical imaging systems. Report of the American Association of Physicists in Medicine (AAPM) Task Group 18; 2005. Accessed April 15, 2021. https://deckard.duhs.duke.edu/~samei/samei_tg18/tg18_files/tg18.pdf

[2] Bevins N , Flynn MJ , Silosky MS , Marsh RM , Walz‐Flannigan AI , Badano A . AAPM Report 270—Display Quality Assurance: The Report of AAPM Task Group 270. American Association of Physicists in Medicine; 2019. Accessed April 15, 2021. https://www.aapm.org/pubs/reports/RPT_270.pdf

[3] Kontrol Af Monitorer Til Røntgen‐Diagnostik . Krav , Vejledninger Og Generelle Anbefalinger for Monitorer Til Brug for Diagnostik. Sundhedsstyrelsen; 2018. Accessed April 15, 2021. https://www.sst.dk/da/udgivelser/2018/kontrol‐af‐monitorer‐til‐roentgendiagnostik

[4] Perry N , Broeders M , de Wolf C , Törnberg S , Holland R , von Karsa L , eds. European Guidelines for Quality Assurance in Breast Cancer Screening and Diagnosis . 4th ed. Office for Official Publications of the European Communities; 2006. Accessed April 15, 2021. https://op.europa.eu/sv/publication‐detail/‐/publication/7945bf8d‐fa10‐4e88‐a781‐9a7c36cf3411

[5] Perry N , Broeders M , de Wolf C , Törnberg S , Holland R , von Karsa L , eds. *European Guidelines for Quality Assurance in Breast Cancer Screening and Diagnosis*—Supplements. 4th ed. Office for Official Publications of the European Union; 2013. 10.2772/13196 18024988

[6] Guidelines for Quality Assurance in Mammography Screening. 4th ed. National Screening Service; 2015. Accessed April 15, 2021. https://www.breastcheck.ie/sites/default/files/guidelines_for_qa_in_mammography_screening_ncss‐pub‐q‐4_rev04.1.pdf

[7] IPEM Report 91: Recommended Standards for the Routine Performance Testing of Diagnostic X‐Ray Imaging Systems. Institute of Physics and Engineering in Medicine; 2005. Accessed April 15, 2021. https://www.ipem.ac.uk/ScientificJournalsPublications/IPEMReportSeries/AvailablePublications.aspx

[8] ACR–AAPM–SIIM . *Technical Standard for Electronic Practice of Medical Imaging*. American College of Radiology; 2017. Accessed April 15, 2021. https://www.acr.org/‐/media/ACR/Files/Practice‐Parameters/elec‐practice‐medimag.pdf 10.1007/s10278-012-9522-2 PMC3553359 22992866

[9] *Guide for Radiation Safety/Quality Assurance Program: Primary Diagnostic Monitors* . New York State Department of Health—Bureau of Environmental Radiation Protection; 2011. Accessed April 15, 2021. https://www.health.ny.gov/environmental/radiological/radiation_safety_guides/docs/pdm_qa.pdf

[10] IEC 62563‐2 ED1 Medical Electrical Equipment—Medical Image Display Systems—Acceptance and Constancy Tests (Draft) . International Electrotechnical Commission; 2020. Accessed April 15, 2021. www.iec.ch

[11] Samei E , Badano A , Chakraborty D , et al. Assessment of display performance for medical imaging systems: executive summary of AAPM TG18 report. Med Phys. 2005;32(4):1205‐1225. 10.1118/1.1861159 15895604

[12] National Electrical Manufacturers Association . *NEMA PS3.14/ISO 12052*: *Digital Imaging and Communications in Medicine (DICOM) Standard—Grayscale Standard Display Function* . NEMA Standards; 2020. http://medical.nema.org/

[13] Sund P . Improving image quality by increasing the amount of light in the reading room. Radiat Prot Dosimetry. 2021:1‐8. Published online April 12, 2021. 10.1093/rpd/ncab047 33839793

[14] Barten PG . Contrast Sensitivity of the Human Eye and Its Effects on Image Quality. SPIE Press; 1999. 10.1117/3.353254

[15] Zele AJ , Cao D . Vision under mesopic and scotopic illumination. Front Psychol. 2015;5:1594. 10.3389/FPSYG.2014.01594 25657632 PMC4302711

[16] Ikushima Y , Yabuuchi H , Morishita J , Honda H . Analysis of dominant factors affecting fatigue caused by soft‐copy reading. Acad Radiol. 2013;20(11):1448‐1456. 10.1016/j.acra.2013.08.013 24119359

[17] Pollard BJ , Chawla AS , Delong DM , Hashimoto N , Samei E . Object detectability at increased ambient lighting conditions. Med Phys. 2008;35(6):2204‐2213. 10.1118/1.2907566 18649449

[18] Pollard BJ , Samei E , Chawla AS , et al. The influence of increased ambient lighting on mass detection in mammograms. Acad Radiol. 2009;16(3):299‐304. 10.1016/j.acra.2008.08.017 19201358

[19] Pollard B , Samei E , Chawla A , et al. The effects of ambient lighting in chest radiology reading rooms. J Digit Imaging. 2012;25(4):520‐526. 10.1007/s10278-012-9459-5 22349990 PMC3389094

[20] Brennan PC , McEntee M , Evanoff M , Phillips P , O'Connor WT , Manning DJ . Ambient lighting: effect of illumination on soft‐copy viewing of radiographs of the wrist. Am J Roentgenol. 2007;188(2):W177‐W180. 10.2214/AJR.05.2048 17242225

[21] McEntee MF , Martin B . The varying effects of ambient lighting on low contrast detection tasks. In: Manning DJ , Abbey CK , eds. Proceedings of SPIE. International Society for Optics and Photonics; 2010:76270N. 10.1117/12.843786

[22] Goo JM , Choi J‐Y , Im J‐G , et al. Effect of monitor luminance and ambient light on observer performance in soft‐copy reading of digital chest radiographs. Radiology. 2004;232(3):762‐766. 10.1148/radiol.2323030628 15273338

[23] Shiiba T , Tanoue N , Tateoka S , Maeda M , Toyofuku F , Morishita J . Effects of ambient‐light correction in luminance measurements of liquid‐crystal display monitors by use of a telescopic‐type luminance meter. Radiol Phys Technol. 2010;3(1):65‐69. 10.1007/s12194-009-0078-x 20821104

[24] Sund P , Båth M , Ungsten L , Månsson LG . Generation of low‐contrast sinusoidal test patterns on a high‐brightness display. J Soc Inf Disp. 2006;14(10):913. 10.1889/1.2372425

[25] Flynn MJ , Tchou P , Accurate measurement of monochrome luminance palettes for the calibration of medical LCD monitors. In: Proceedings of SPIE. Vol. 5029. International Society for Optics and Photonics; 2003:438‐448.

[26] Leek MR . Adaptive procedures in psychophysical research. Percept Psychophys. 2001;63(8):1279‐1292. 10.3758/BF03194543 11800457

[27] Pelli DG , Bex P . Measuring contrast sensitivity. Vision Res. 2013;90:10‐14. 10.1016/j.visres.2013.04.015 23643905 PMC3744596

[28] Klein SA . Measuring, estimating, and understanding the psychometric function: a commentary. Percept Psychophys. 2001;63(8):1421‐1455. 10.3758/BF03194552 11800466

[29] Wichmann FA , Hill NJ . The psychometric function: I. Fitting, sampling, and goodness of fit. Percept Psychophys. 2001;63(8):1293‐1313. 10.3758/BF03194544 11800458

[30] Karmali F , Chaudhuri SE , Yi Y , Merfeld DM . Determining thresholds using adaptive procedures and psychometric fits: evaluating efficiency using theory, simulations, and human experiments. Exp Brain Res. 2016;234(3):773‐789. 10.1007/s00221-015-4501-8 26645306 PMC4831214

[31] Madigan R , Williams D . Maximum‐likelihood psychometric procedures in two‐alternative forced‐choice: evaluation and recommendations. Percept Psychophys. 1987;42(3):240‐249. 10.3758/BF03203075 3671049

[32] Treutwein B . Adaptive psychophysical procedures. Vision Res. 1995;35(17):2503‐2522. 10.1016/0042-6989(95)00016-X 8594817

[33] García‐Pérez MA . Forced‐choice staircases with fixed step sizes: asymptotic and small‐sample properties. Vision Res. 1998;38(12):1861‐1881. 10.1016/S0042-6989(97)00340-4 9797963

[34] Efron B . The Jackknife, the Bootstrap and Other Resampling Plans. Society for Industrial and Applied Mathematics; 1982. 10.1137/1.9781611970319

[35] Maloney LT . Confidence intervals for the parameters of psychometric functions. Percept Psychophys. 1990;47(2):127‐134. 10.3758/BF03205977 2304811

[36] Wichmann FA , Hill NJ . The psychometric function: I. Bootstrap‐based confidence intervals and sampling. Percept Psychophys. 2001;63(8):1314‐1329. 10.3758/BF03194545 11800459

[37] Barten PGJ , Formula for the contrast sensitivity of the human eye. In: Miyake Y , Rasmussen DR , eds. Proceedings of SPIE 5294, Image Quality and System Performance. Vol. 5294. International Society for Optics and Photonics; 2003:231‐238. 10.1117/12.537476

[38] Baxter B , Ravindra H , Normann RA . Changes in lesion detectability caused by light adaptation in retinal photoreceptors. Invest Radiol. 1982;17(4):394‐401. Accessed October 21, 2014. http://www.ncbi.nlm.nih.gov/pubmed/7129821 7129821 10.1097/00004424-198207000-00017

[39] Flynn MJ , Kanicki J , Badano A , Eyler WR . High‐fidelity electronic display of digital radiographs. RadioGraphics. 1999;19(6):1653‐1669. 10.1148/radiographics.19.6.g99no081653 10555680

[40] Sund P , Månsson LG , Båth M . Development and evaluation of a method of calibrating medical displays based on fixed adaptation. Med Phys. 2015;42(4):2018‐2028. 10.1118/1.4915531 25832092

[41] Radonjić A , Allred SR , Gilchrist AL , Brainard DH . The dynamic range of human lightness perception. Curr Biol. 2011;21(22):1931‐1936. 10.1016/j.cub.2011.10.013 22079116 PMC3244211

[42] Paulsson L , Sjostrand J . Contrast sensitivity in the presence of a glare light: theoretical concepts and preliminary clinical studies. Invest Ophthalmol Vis Sci. 1980;19(4):401‐406. Accessed April 15, 2021. https://iovs.arvojournals.org/article.aspx?articleid=2159058 7358491

[43] Abrahamsson M , Sjostrand J . Impairment of contrast sensitivity function (CSF) as a measure of disability glare. Invest Ophthalmol Vis Sci. 1986;27(7):1131‐1136. Accessed April 15, 2021. https://iovs.arvojournals.org/article.aspx?articleid=2177597 3721791

